# A Randomized, Placebo-Controlled, Pilot Clinical Trial to Evaluate the Effect of Supplementation with Prebiotic Synergy 1 on Iron Homeostasis in Children and Adolescents with Celiac Disease Treated with a Gluten-Free Diet

**DOI:** 10.3390/nu10111818

**Published:** 2018-11-21

**Authors:** Klaudia Feruś, Natalia Drabińska, Urszula Krupa-Kozak, Elżbieta Jarocka-Cyrta

**Affiliations:** 1 Department of Pediatrics, Gastroenterology and Nutrition, Collegium Medicum Faculty of Medicine, University of Warmia & Mazury, Oczapowskiego 2 Str., 10-719 Olsztyn, Poland; klaudiaferus@o2.pl; 2 Department of Chemistry and Biodynamics of Food, Institute of Animal Reproduction and Food Research of Polish Academy of Sciences, Tuwima 10 Str., 10-748 Olsztyn, Poland; n.drabinska@pan.olsztyn.pl (N.D.); u.krupa-kozak@pan.olsztyn.pl (U.K.-K.)

**Keywords:** celiac disease, iron deficiency anemia, gluten-free diet, inulin, prebiotics, iron absorption, hepcidin

## Abstract

Iron deficiency anemia (IDA) occurs in 15–46% of patients with celiac disease (CD), and in some cases, it may be its only manifestation. Studies in animal models have shown that prebiotics, including inulin, may help to increase intestinal absorption of iron. The aim of this study was to evaluate the effect of a prebiotic, oligofructose-enriched inulin (Synergy 1), on iron homeostasis in non-anemic children and adolescents with celiac disease (CD) in association with a gluten-free diet (GFD). Thirty-four CD patients (4–18 years old) were randomized into two groups receiving Synergy 1 (10 g/day) or a placebo (maltodextrin) for three months. Before and after intervention, blood samples were collected from all patients for assessment of blood morphology, biochemical parameters and serum hepcidin concentration. We found that serum hepcidin concentration after the intervention was significantly decreased by 60.9% (*p* = 0.046) in the Synergy 1 group, whereas no significant difference was observed in the placebo group. No differences in morphological and biochemical blood parameters (including ferritin, hemoglobin and C-reactive protein (CRP)) were observed after intervention in either group. Given that hepcidin decrease may improve intestinal iron absorption, these results warrant further investigation in a larger cohort and especially in patients with IDA.

## 1. Introduction

Celiac disease (CD) is a small intestine enteropathy that is triggered by the ingestion of storage proteins (gluten) from wheat, barley or rye. It occurs in genetically predisposed individuals at any age, at a frequency of 1:100 [1,2,3,4]. Characteristic features of CD include a massive lymphocytic infiltration of the lamina propria and atrophy of intestinal villi. Consequently, there is a significant reduction of the intestinal absorption surface, leading to malabsorption of macro- and micronutrients [2,4,5].

Iron deficiency anemia (IDA) is a common finding in children and adults with CD, with an estimated prevalence at diagnosis between 15% and 46% [6]. Anemia may accompany the intestinal presentation of CD, but it can also be the only manifestation of the disease. As such, the possibility of CD should be considered in patients with refractory anemia after other possible causes have been excluded [7]. Iron absorption and distribution is tightly controlled. Hepcidin, a 25-amino-acid peptide hormone produced in the liver, is a central regulator of systemic iron homeostasis. Iron deficiency and hypoxia can decrease hepcidin production, while the pro-inflammatory cytokine IL-6 increases hepcidin expression. Increased serum levels of hepcidin contribute to anemia in chronic diseases [8,9].

Previous studies have shown that the main cause of IDA in CD patients is the limited iron absorption, as a consequence of chronic damage of the intestinal mucosa [6]. Other authors have highlighted the role of chronic mucosal inflammation [10], and the presence of mutations in genes encoding proteins involved in iron absorption [11,12].

The only known therapy for celiac disease is a lifelong gluten-free diet (GFD). Adherence to a GFD leads to recovery of the intestinal mucosa, thereby normalizing nutrient absorption. In most patients, a 6-month period is adequate for nutritional absorption to improve [13]. Normalization of iron and hemoglobin levels depends on the severity of the disease at presentation, compliance to GFD, and bioavailability of dietary iron. In the majority of patients, anemia resolves after approximately one year of a GFD, but persistent IDA is observed in about 8% of patients despite a GFD and even up to 20.5% according to some reports [14,15]. Evaluation of serum CD-associated antibodies, such as anti-tissue transglutaminase antibodies, and the assessment of clinical symptoms, are the most commonly used methods to assess CD patients during follow-up. However, these antibodies often decrease and/or disappear regardless of histological healing and GFD adherence [16]. In one study, complete histological recovery after one year of well-followed GFD in adults was only obtained in 66% of patients [17]. Moreover, GFD itself may result in further deficiencies, including fibre, B vitamins, iron, and trace minerals [18,19]. Decreased iron intake while following a GFD has been reported [20]. All abovementioned clinical circumstances can influence the availability and absorption rate of iron and result in prolonged iron deficiency. Thus, additional safe and easily accepted therapeutic options to improve the iron status in CD patients are needed.

Prebiotics, typically oligosaccharides, such as fructo- and galactooligosaccharides (FOS and GOS) or inulin, have been shown to improve bioavailability of minerals, and to enhance iron absorption in animal studies [21,22].

The aim of this study was to evaluate the effect of oligofructose-enriched inulin (Synergy 1) on iron homeostasis in CD children following a GFD. We posited that Synergy 1 supplementation would result in an improvement of blood morphology and other parameters relative to iron homeostasis.

## 2. Materials and Methods

### 2.1. Study Design

We performed a single-center, randomized, placebo-controlled, double-blind study in patients diagnosed with CD and treated with a GFD. The intervention consisted of introducing oligofructose-enriched inulin (Synergy 1) into the diet for 12 weeks. We assessed the impact of the intervention on nutritional status, morphological and biochemical blood parameters and gut microbiota. Details of the study protocol have been previously described by Krupa-Kozak et al. [23]. Results regarding nutritional status, gut microbiota composition, and short-chain fatty acids concentration in the stool have been previously reported elsewhere [24].

### 2.2. Participants Selection

Participants were enrolled among consecutive patients with celiac disease, aged 4–18 years, treated with a gluten-free diet for at least 6 months prior to enrolment, treated and followed-up at the Department of Paediatrics, Gastroenterology and Nutrition Medical Faculty of University of Warmia and Masuria in Children’s Hospital, Olsztyn, Poland. CD was diagnosed according to criteria created by the European Society for Paediatric Gastroenterology, Hepatology and Nutrition (ESPGHAN 2012 criteria) [25]. All patients had positive (≥8 AU/mL) anti-transglutaminase 2 antibodies at the time of diagnosis. To confirm the diagnosis, endoscopy with small bowel biopsies was performed in all patients and the specimens were interpreted according to the Marsh criteria [25]. Among the 96 patients who met the inclusion criteria (Table 1), a consent to participate in the study was obtained for 34 patients.

### 2.3. Ethics

Parents and caregivers were informed about potential benefits and risks and signed a written consent form during the enrollment visit. Experimental design and all procedures were approved by the Bioethics Committee of the Faculty of Medical Sciences of the University of Warmia and Mazury in Olsztyn (permission No. 23/2015 of 16 June 2015). The study was registered in the ClinicalTrials database (NCT03064997) [27].

### 2.4. Intervention

Patients (*n* = 34) were randomly assigned to the placebo group (*n* = 16) or the Synergy 1 group (*n* = 18) [23]. The intervention lasted 3 months. Participants in the control group received maltodextrin (7 g orally/day; Maltodextrin DE 20, Hotrimex, Konin, Poland), while participants in the examination group received oligofructose-enriched inulin (10 g orally/day; Orafti^®^ Synergy 1, Beneo, Tienen, Belgium). Patients, parents/caregivers, and all investigators except N.D. (who was in charge of the treatment distribution) were blinded to the allocated experimental group. Maltodextrin was the placebo of choice, as it is digested in the small intestine and thus does not exert local effects in the colon, contrarily to prebiotics. During the study, patients were required to record adherence to supplementation, side effects, if any, and the intake of other substances, i.e., antibiotic, probiotic, or prebiotic. The nutritional value of the diet during study and adherence to GFD were monitored using a validated food frequency questionnaire (FFQ-6) [28].

### 2.5. Sample Collection

Blood samples were collected from all participants at two time points: before and after the intervention. Complete blood count and biochemical parameters (C-reactive protein (CRP) and ferritin) were analyzed according to standard procedures of the hospital laboratory, as previously described [23]. Serum hepcidin levels were measured using a commercial ELISA kit (FRG Instruments GmbH, Nuremberg, Germany).

### 2.6. Statistical Analysis

All below analyses were performed in duplicate and the data were analyzed using the Statistica 12 software (StatSoft, Tulsa, OK, USA). A difference with a *p*-value < 0.05 was considered statistically significant. Normality of quantitative variables was tested by the Shapiro–Wilk W test. Quantitative variables with a normal distribution were expressed as mean ± SD, while quantitative variables which showed a non-normal distribution were expressed as a median (P25-P75). Differences in characteristics between groups were tested with the parametric Student’s *t*-test or the non-parametric Mann–Whitney U test, as appropriate. Differences within groups before and after intervention were determined with the Student’s *t*-test for paired samples or the Wilcoxon signed-rank test, as appropriate.

## 3. Results

### 3.1. Study Population

Thirty-four children and adolescents (mean age 10 years; 62% females; all anthropometric details summarized in Table 2), were included in the study. The duration of the GFD prior to enrolment ranged between seven months to nine years but showed no significant difference between Synergy 1 and the placebo group (*p* = 0.608). Thirty patients completed the study (88.2%), while four children were excluded from the analysis due to non-compliance in the test protocol.

The safety profile and side effects of Synergy 1 in this trial have been previously described [24]. Briefly, no severe side effects were noted during the three-month intervention with Synergy 1 and there was no significant difference in the frequency of reported symptoms between the two experimental groups. The levels of anti-tissue transglutaminase antibodies (tTGA) were measured before and after intervention. In all patients, tTGA titers before and after intervention were within the recommended level (<8.0 AU/mL). All patients had adequate adherence to GFD according to the FFQ-6 questionnaire.

### 3.2. Morphological and Biochemical Parameters of Blood

Morphological and biochemical blood parameters at baseline were comparable between the Synergy 1 and the placebo group. No statistically significant difference in those parameters was observed before and after intervention in either of the two experimental groups (Table 3).

### 3.3. Hepcidin

**Figure 1:**
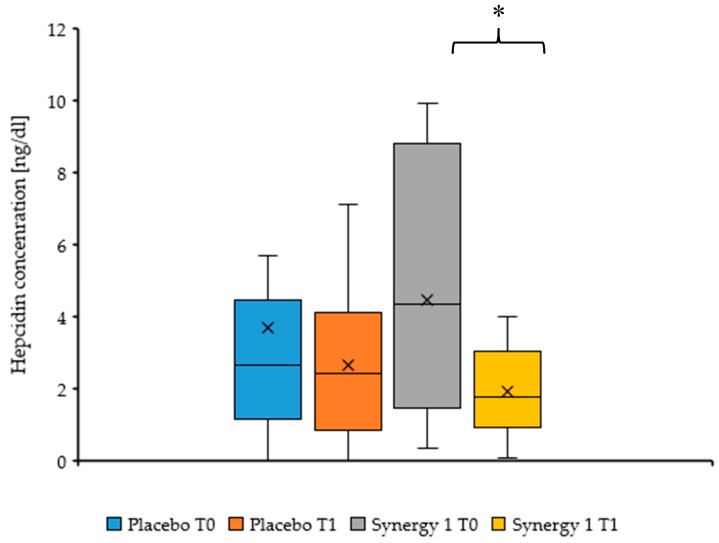
Serum hepcidin concentration before (T0) and after (T1) intervention, expressed as a median (cross) (P25-P75) (box). * *p* = 0.046.

Serum hepcidin concentrations at baseline (T0) were comparable between the two groups (*p* = 0.547). Hepcidin levels in the Synergy 1 group were significantly lower after 3-months intervention than at baseline (median: 1.73 (1.31–3.14) versus 4.42 (1.89–8.64), respectively; *p* = 0.046), accounting for a 60.9% decrease. Conversely, no significant difference in hepcidin concentration was observed between T1 and T0 in the placebo group (median: 2.43 (0.91–3.87) versus 2.99 (1.23–5.09), respectively) (Figure 1). There was no significant difference between the Synergy 1 and placebo group after the intervention (*p* = 0.645).

## 4. Discussion

To our knowledge, this is the first prospective, randomized, placebo-controlled, double-blind study of the effects of oligofructose-enriched inulin (Synergy 1) on iron homeostasis in CD patients treated with a GFD. Our key finding was a significant decrease in plasma hepcidin concentration after 3 months of treatment with Synergy 1 (10 g daily), whereas no such effect was observed in the placebo group. Hepcidin downregulates duodenal iron absorption and decreases iron storage release by modulating cellular export via ferroportin [11]. Hepcidin production disorders result in impaired iron homeostasis: Hepcidin deficiency may cause iron overload, while excess is associated with IDA [8,9]. Thus, the observed decrease in hepcidin levels upon the Synergy 1 treatment could potentially help improve iron absorption in CD children and adolescents.

To confirm this hypothesis, a positive effect of Synergy 1 on ferritin levels would need to be demonstrated. Plasma ferritin concentration is the most sensitive indicator of iron storage capacity in IDA. In our study, the Synergy 1 treatment did not alter ferritin or haemoglobin levels. However, in patients with normal iron stores, ferritin levels are finely regulated to avoid excessive iron absorption and accumulation in the organism [7]. Given that the present study was conducted on non-anemic children and adolescents, the potential effect of Synergy 1 on ferritin and on haemoglobin levels cannot be properly evaluated. Further prospective studies are warranted, focusing on CD children with IDA and especially on those with refractory IDA, to verify whether Synergy 1 can indeed increase iron absorption and whether it could have a clinical benefit in this setting. As a secondary finding, our results show that Synergy 1 does not cause excessive iron accumulation or iron deficiency in non-anemic CD patients, thus supporting a safe profile of this prebiotic in regards to iron homeostasis in non-anemic individuals.

One possible explanation of the observed decrease in hepcidin is the potential anti-inflammatory effect of prebiotics, which has been previously reported in animal models. In a study by Marciano et al. [22], supplementation of anemic growing rats with oligofructose, but not with inulin, led to decreased TNF-α, IL-6 and IL-10 expression in the cecum and to a decrease in urinary hepcidin. In another study, inulin and oligofructose supplementation led to a downregulation of pro-inflammatory genes in colonic tissue of young anemic pigs [29]. Although the Synergy 1 treatment did not influence CRP levels in our study, pro-inflammatory cytokines were not measured.

The beneficial role of prebiotics on iron absorption could have important clinical implications, but so far, results from different model systems have been discrepant. Many animal studies suggest a positive effect [22,29,30]. For example, in rats, a beneficial effect of FOS supplementation on iron absorption was observed in both iron-deficient animals [22,31] and in growing rats with a normal iron status [32]. In vitro experiments on the human cell line Caco-2, a widely-used model for studying absorptive proprieties of the intestinal mucosa, have yielded inconsistent results: in two studies, prebiotics did not improve iron bioavailability from milk- or soy-based yogurts [33,34], while in two other studies, iron bioavailability from iron-fortified cereal biscuits [35] and from the commercial Young Child Formula^®^ [36] were significantly improved by prebiotic supplementation. In humans, no positive results are available to date. In healthy men aged 20–30 years, iron absorption measured using a stable isotope technique was 20% higher in individuals supplemented daily with FOS (15 g/day for 21 days) than in the control group, but the results did not reach statistical significance [37]. In women with anemia, supplementation with 20 g of inulin per day for four weeks did not cause an increase of iron absorption, although changes in gut microbiota composition and a decrease of fecal pH were observed [38].

Although our work reveals a potential link between prebiotic supplementation and hepcidin levels, prebiotics may also enhance iron absorption in other ways, including a direct effect on iron transporter expression [22] and their potential to decrease systemic inflammation [22,29]. Moreover, fermentation of indigestible oligosaccharides increases the production of fatty acids by *Bifidobacteria* spp. and lowers the fecal pH, which in turn can improve iron solubility and enhance its absorption [21,38]. The potential effect of prebiotics on iron status could also be a more complex process, affecting not only absorption but also the stage of transfer, storage, and recycling [22,29,30].

Healing of the intestinal mucosa is a critical step towards recovering normal absorption of macro- and micro-nutrients. However, CD patients with anemia usually show a more severe enteropathy than non-anemic patients and their intestinal mucosa may take longer to heal [38].

Our study has some limitations, including a small cohort size, inclusion of patients within a wide age range, and a relatively short intervention duration. Thus, these findings need to be validated on a larger cohort, including patients with IDA, and measuring additional parameters, such as pro-inflammatory cytokines, with the potential to elucidate the mechanisms of hepcidin changes in this setting. Intestinal histological healing in children after GFD has also been shown to occur earlier and to a greater extent than in adults [39]. Thus, further research is needed to establish the potential role of Synergy-1 in iron hemostasis in adult CD patients.

## 5. Conclusions

We have previously shown that oligofructose-enriched inulin (Synergy 1) was a safe and well-tolerated prebiotic in children and adolescents with CD in association with a GFD. Here, we found that a three-month intervention with Synergy 1 (10 g orally/day) led to a significant decrease of serum hepcidin concentrations by 60.9% (*p* = 0.046) in those patients, whereas no significant difference was observed in the placebo group. Given that hepcidin decrease may improve iron absorption, these promising results warrant further investigation in a larger cohort, including patients with iron deficiency anemia, who represent the potential target group for this type of treatment.

## Figures and Tables

**Table 1:** Participant selection criteria.

Inclusion Criteria	Exclusion Criteria
Diagnosed Celiac Disease Gluten-free diet for at least 6 months Age: 4–18 years old Normalization of Tissue Transglutaminase Antibody (TTGA) level Written consent from parents/caregivers	Iron deficiency anemia ^1^ Iron deficiency ^2^ Immunoglobulin A (IgA) deficiency Treatment with oral formulas in the 2 months prior to the study Therapy by antibiotics or probiotics/prebiotics in the 2 months prior to the study Chronic inflammatory disorders

^1^ Iron deficiency anemia was defined as a hemoglobin level below WHO range for sex and age. ^2^ iron deficiency was defined as a ferritin level <12 ng/mL [26].

**Table 2:** Participant anthropometric data.

	Total Sample		Intervention Group (Synergy 1)		Placebo Group (Maltodextrin)	
N	30		17 (56.6%)		13 (63.4%)	
Gender (G–girls, B–boys)	G = 18 (60%)		G = 10 (58.8%)		G = 8 (61.5%)	
	B = 12 (40%)		B = 7 (41.2%)		B = 5 (38.5%)	
Age (years)	4–18		4–18		4–16	
	Average = 10		Average = 10		Average = 10	
	**T0 ^a^**	**T1 ^b^**	**T0**	**T1**	**T0**	**T1**
Weight (kg)	15.0–78.0	15.7–77.5	15.0–78.0	15.7–77.5	16.3–66.8	17.0–71.5
	Av = 35.8	Av = 37.6	Av = 35.8	Av = 37.6	Av = 33.7	Av = 36.2
Height (cm)	103.0–183.0	104.5–184.5	104.5–183.0	108.0–184.5	103.0–172.0	104.5–172.6
	Av = 139.6	Av = 141.4	Av = 141.5	Av = 142.4	Av = 137.1	Av = 139.7
BMI (kg/m^2^)	12.5–28.4	12.7–29.0	12.5–23.5	12.7–23.6	13.7–28.4	13.4–29.0
	Av = 17.1	Av = 17.3	Av = 17.1	Av = 17.3	Av = 17.0	Av = 17.3

^a^ T0—baseline; ^b^ T1—after three-month intervention.

**Table 3:** Morphological and biochemical parameters before (T0) and after (T1) the intervention, expressed as mean ± SD.

Morphology Parameters	Synergy 1 Group		Placebo Group		Synergy 1: T0 vs. T1 ^1^ (*p* Value)	Placebo: T0 vs. T1 ^1^ (*p* Value)	T1: Synergy 1 vs. Placebo (*p* Value)
	T0	T1	T0	T1			
Red Blood Cell (10^6^/mm^3^)	4.63 ± 0.37	4.69 ± 0.34	4.58 ± 0.37	4.57 ± 0.34	0.274	0.851	0.359
Hemoglobin (g/dL)	13.22 ± 0.99	13.13 ± 1.09	13.12 ± 0.99	12.89 ± 1.09	0.912	0.297	0.565
Hematocrit (%)	39.11 ± 2.95	39.65 ± 3.22	38.94 ± 2.95	38.93 ± 3.22	0.314	0.838	0.559
Mean Cell Volume (µm^3^)	84.50 ± 4.18	84.63 ± 4.33	85.19 ± 4.18	84.92 ± 4.33	1.000	0.779	0.283
Mean Cell Hemoglobin (pg)	28.48 ± 1.53	27.65 ± 1.17	28.65 ± 1.53	28.13 ± 1.17	0.139	0.052	0.102
Red Blood Cell Distribution Width (%)	12.64 ± 0.71	12.91 ± 0.92	12.99 ± 0.71	13.22 ± 0.92	0.247	0.308	0.695
Platelets (10^3^/mm^3^)	290.28 ± 64.15	314.63 ± 55.51	301.38 ± 64.15	315.77 ± 55.51	0.299	0.197	0.957
White Blood Cell (10^3^/mm^3^)	6.29 ± 1.64	6.57 ± 1.78	6.59 ± 1.64	6.65 ± 1.78	0.721	0.844	0.283
**Biochemical parameters**							
C-reactive protein (CRP) (mg/dL)	0.14 ± 0.08	0.11 ± 0.07	0.10 ± 0.08	0.12 ± 0.07	0.582	0.100	0.660
Ferritin (ng/mL)	25.78 ± 14.48	22.94 ± 13.94	27.62 ± 14.48	23.08 ± 13.94	0.507	0.107	0.742

^1^ Comparison within groups using Student’s *t*-test or the Wilcoxon test, as appropriate.
